# Electrochemistry-stimulated environmental bioremediation: Development of applicable modular electrode and system scale-up

**DOI:** 10.1016/j.ese.2020.100050

**Published:** 2020-06-26

**Authors:** Ai-Jie Wang, Hong-Cheng Wang, Hao-Yi Cheng, Bin Liang, Wen-Zong Liu, Jing-Long Han, Bo Zhang, Shu-Sen Wang

**Affiliations:** a School of Civil & Environmental Engineering, Harbin Institute of Technology (Shenzhen), Shenzhen, 518055, PR China; b Key Laboratory of Environmental Biotechnology, Research Center for Eco-Environmental Sciences, Chinese Academy of Sciences, Beijing, 100085, PR China

**Keywords:** Environmental bioremediation, Bio-electrochemical systems (BESs), Electrode modular, Scaling-up, Intergradation system

## Abstract

Bioelectrochemical systems (BESs) have been studied extensively during the past decades owing primarily to their versatility and potential in addressing the water-energy-resource nexus. In stark contrast to the significant advancements that have been made in developing innovative processes for pollution control and bioresource/bioenergy recovery, minimal progress has been achieved in demonstrating the feasibility of BESs in scaled-up applications. This lack of scaled-up demonstration could be ascribed to the absence of suitable electrode modules (EMs) engineered for large-scale application. In this study, we report a scalable composite-engineered EM (total volume of 1 m^3^), fabricated using graphite-coated stainless steel and carbon felt, that allows integrating BESs into mainstream wastewater treatment technologies. The cost-effectiveness and easy scalability of this EM provides a viable and clear path to facilitate the transition between the success of the lab studies and applications of BESs to solve multiple pressing environmental issues at full-scale.

## Highlights

• Sandwich-corrugated based applicable electrode module (EM) was developed.
• EM were optimized designed in 2D material and 3D configuration.
• The integration strategies for EM and anaerobic process were introduced.
• Future perspectives of EM application in environmental bioremediation was expected.

## 1 Introduction

Bioelectrochemical systems (BESs) are a suite of electrochemical devices that rely on microorganisms to catalyze the redox reactions on the electrodes [1,2]. Over the past two decades, the functions of BESs have been extensively expanded from bioelectricity generation [3,4] to biohydrogen production, enhanced methane production [5], desalination [6,7], and accelerated pollutants reduction [[8], [9], [10]]. Such versatility, combined with their seemingly simple configurations, has garnered considerable research attention for BESs, as these systems also have the potential to solve the water-energy-resource nexus [11].

In stark contrast to the remarkable advancements that have been achieved in bench-scale BESs studies, relatively minimal progress has been made in demonstrating the engineering feasibility of BESs at pilot- or full-scale. There are several reasons that hinder the successful transformation of bench-scale BES studies to viable full-scale devices [12]. Among these, the lack of electrodes/electrode modules (EMs) that are suitable for large-scale application is arguably the single largest obstacle [13]. Compared to the electrodes for lab-scale BES applications, the electrode/EMs for potential scale-up BESs must have additional features such as structural rigidity, electrical conductivity across the electrode/EM, and be conducive to mass transfer.

To date, different types of electrodes have been tested in lab-scale BESs studies [[14], [15], [16], [17], [18], [19], [20], [21]]. These electrodes typically have a relatively simple configuration with desirable features for (micro)liter-scale reactors. However, the less-engineered configurations of these bench-scale electrodes have inherent flaws that render it impossible to construct full-scale BESs using these electrodes. For example, a carbon brush is the most widely used anode electrode in bench-scale BESs studies [[22], [23], [24]]; however, its low mechanical strength and relatively high volumetric cost (i.e., the cost of electrodes per unit electrode volume) make it impossible to use as an electrode in full-scale applications. In addition to the carbon-brushes, stainless steel (SS) mesh is another commonly used electrode in lab-scale studies [[25], [26], [27], [28], [29]]. Although a SS mesh could be processed using readily available techniques and purchased in bulk quantity from the marketplace, its relatively low specific surface area and biocompatibility prevent an SS mesh electrode from being used solely in electrodes for large-scale BESs [30,31]. Ideally, the electrode configurations that are suitable to be used in large-scale BESs should include the features of cost-effectiveness, high mechanical strength, large specific surface area, high biocompatibility, and acceptable electrical conductivity [13]; however, to the best of our knowledge, no such electrode configuration has been reported to date.

In addition to electrode configuration, another indispensable component in large-scale BESs is the power management system (PMS) [32]. It is expected that large-scale BESs would require multiple electrodes/EMs to operate simultaneously to meet the treatment demand. In fact, for BESs consisting of multiple electrodes or stacked reactors, it is not uncommon to have a situation wherein the well-performing electrodes/reactors are negatively influenced by the sub-optimized performing electrodes/reactors. Under such a circumstance, it is then of significant importance to have the ability to monitor and control the individual electrodes to ensure the overall performance of the scaled-up system. Different PMSs for lab-scale BESs have been developed to achieve the goal of regulating multiple electrode/reactors in complex BESs. For example, a PMS based on a field-effect transistor has been reported for optimizing the growth condition of a bioanode during the startup period of a stacked microbial fuel cell. Whereas this PMS could protect and significantly reduce the startup period of bioanodes, it should be noted that only three lab-scale microbe fuel cells (MFCs) were included in the tested stacked BES, which left the question of connecting, monitoring, and regulating multiple electrodes in scaled-up BESs unanswered.

In this study, we propose a novel composite EM designed specifically for application in scaled-up BESs. This novel EM is fabricated primarily with SS and carbon felt and was tested for its applicability in several enlarged BES reactors with varying sizes and functions. The key limitations associated with scaling up, such as electrode material and configuration, PMS, the arrangement of multiple modules, and the cost analysis [33], are accorded specific attention in this study. Moreover, the challenges and niche applications of the proposed newly designed EMs integrating with current environmental bioremediation technologies (anaerobic treatment, advanced treatment process, constructed wetlands) to hybrid processes are also discussed.

## 2 Development of applicable EM for BESs

### 2.1 Selection and modification of raw electrode materials

Different materials have been used to fabricate electrodes for BESs at the lab- and pilot-scale [34]. Among these materials, we chose carbon and SS as the raw materials to fabricate the electrodes for a scaled-up BESs for the following two reasons: firstly, these two materials have been extensively studied in lab- and pilot-scale studies, and have long been recognized for their electrical conductivity [35]; secondly, the processing techniques to fabricate electrodes using these two materials are readily available, which makes it possible to engineer the configurations of the EMs [26,27].

**Fig. 1:**
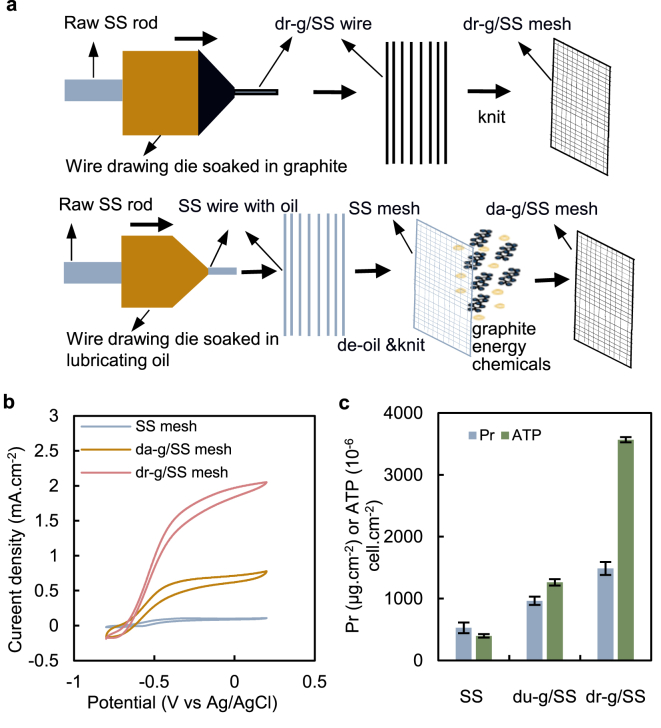
(a) Graphite coating SS mesh manufacturing process, one-step modification process (above), traditional process (below); (b) cyclic voltammograms (scan rate: 10 mV/s) of electrode materials. (c) the biocompatibility of electrode materials.

However, the reductive capability of cultivating well-performing exoelectrogens is the challenge in the process of magnifying naked SS for an electrode. To facilitate the exoelectrogens growth on SS, we propose a one-step modification process for depositing graphite on a SS wire surface at the time of the SS wire processing (Fig. 1a). This surface modification method is energy-efficient and environmentally friendly, as depositing the graphite in situ utilizes the waste heat generated by the SS wire production process, without chemical agent input. The electrocatalytic activity of the drawn graphite-coated stainless steel (dr-g/SS) was 20 and 7 times greater than the SS and daubed graphite-coated stainless steel (da-g/SS), respectively (Fig. 1b). Moreover, the biocompatibility (adenosine triphosphate (ATP) content and protein of the electrode biofilm) of the dr-g/SS was observed to be considerably greater than the da-g/SS and SS (Fig. 1c). Based on the graphite used, the cost involved in the one-step graphite coating of SS is approximately 0.57 $.m^-2^-SS mesh, which is considerably less than previous methods such as the deposition of carbon nanostructures by acetylene gas treatment [36], pyrolysis of pyridine for growing nitrogen-doped carbon nanofibers [37], flame oxidation to generate iron oxide nanoparticles [31], deposition carbon layer by α-D-glucose impregnation, caramelization, and pyrolysis [30].

### 2.2 Engineering and fabrication of EMs

Having determined the raw materials for individual electrodes, we next shifted our focus to the engineering of EMs. The ideal EMs of a scaled-up BESs should have the properties of biocompatibility, electrical conductivity, a large possessing surface area, structural rigidity, and be conducive to mass transfer [31,35,38,39]. Whereas the first three properties can be attained using suitable electrode materials, the last two properties, which are of critical importance for application in scaled-up BESs, can only be achieved through the proper engineering of the EMs.

**Fig. 2:**
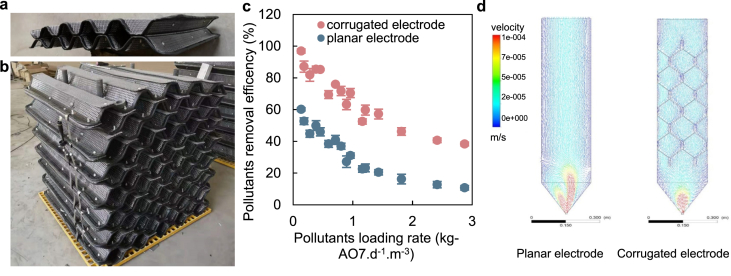
(a) photograph of a pair of sandwich-corrugated electrodes; (b) photograph of the main part for a sandwich-corrugated EM; (c) pollutant-removal performance of corrugated electrode and planar electrode; (d) water flow velocity distributions.

To address the engineering demand of scaled-up BESs, we developed a sandwich corrugated-type EM, using g-SS mesh and carbon felt as the raw materials. The sandwich-corrugated module was composed of individual electrodes comprised of carbon felt and corrugated g-SS mesh (Fig. 2a–b). The carbon felt was used primarily as a substratum for the growth of the exoelectrogens; the corrugated g-SS mesh was employed as a current collector and to provide the structural rigidity required for the EMs. In each individual electrode, the carbon felt was sandwiched between two pieces of SS mesh with matching corrugates to form one electrode. Another electrode, with the same configuration and opposite corrugate pattern, was used as the counter electrode to form a pair of electrodes for the EMs. The channels formed by placing corrugated mesh with opposite patterns were intended to facilitate the flow of influent. Care was exercised to electrically isolate the two electrodes next to each other by insulating the frames of the SS mesh, and inserting insulation pads between the bolt joints. A single EM was formed by stacking the corrugated electrodes in a pair-wise manner.

Our previous studies demonstrated that the corrugated configuration can dramatically improve the degradation of refractory pollutant (such as azo dye) [25,40]. Six sets of corrugated electrodes with different angles and electrode spacings were installed in a lab-scale anaerobic reactor to treat the mixed source of domestic and industrial wastewaters containing organic dyes (Fig. 2c). Pollutant-removal improvement can be achieved by simply folding a conventional planar electrode to be a corrugated type without additional material cost. Compared to a conventional planar electrode, the corrugated electrode demonstrated a greater volumetric decolorization rate (255% of planar electrode) and reduced start-up time (reduced from 5 days to 2 days) [25]. These enhancements were proved to prolong the mean residence time of the pollutants and change the flow pattern closer to the plug flow (Fig. 2d). The optimal configuration was observed with a 40° folding angle and 2 mm electrode spacing when considering the efficiency and economic effects comprehensively.

### 2.3 Power management system

**Fig. 3:**
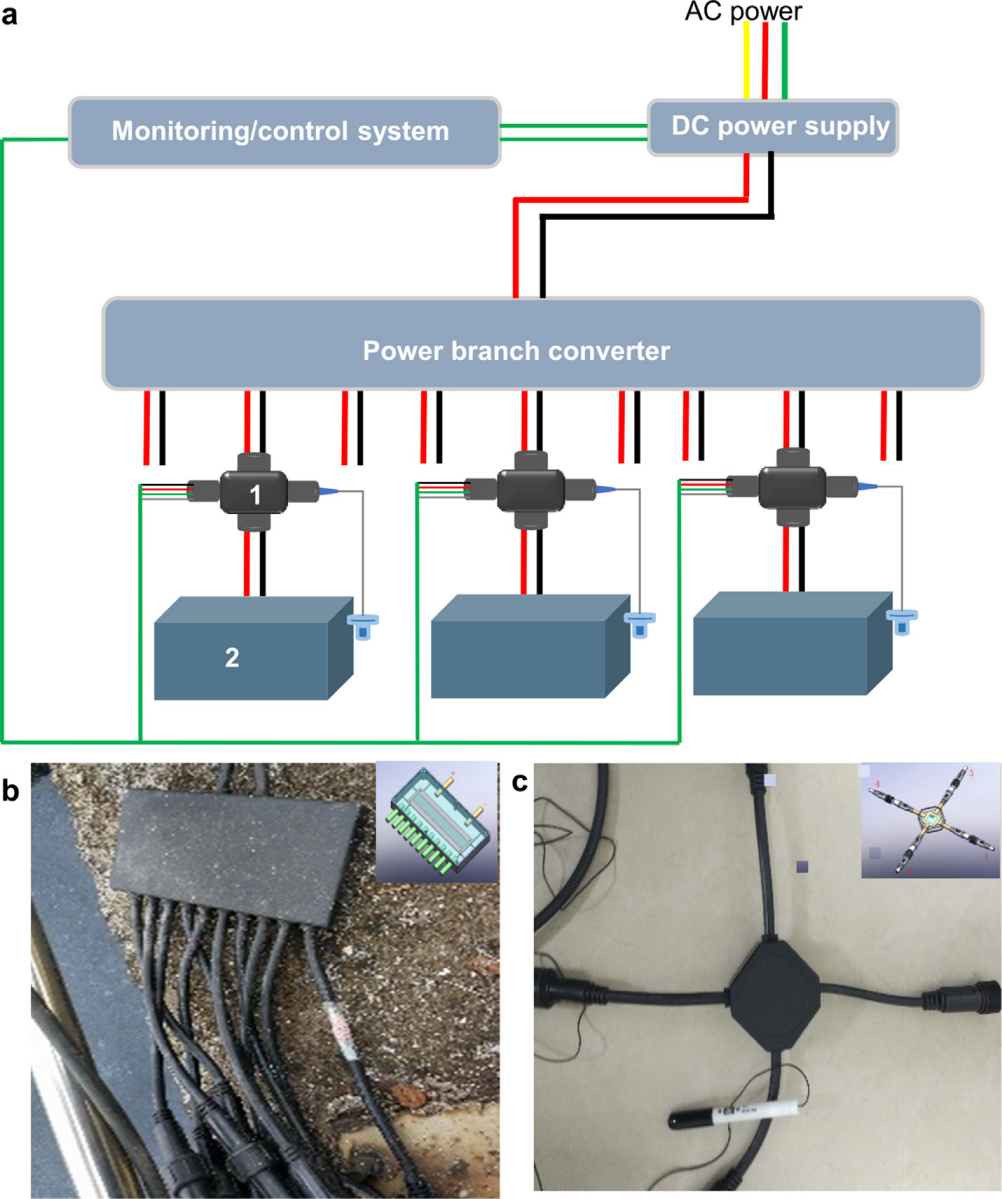
(a) conceptual global diagram of PMS: “1” represent signal transmission system, “2” represents electrode component of applicable electrode module; (b) photograph and conceptual diagram of power branch converter; (c) photograph and conceptual diagram of signal transmission system.

As mentioned above, the PMS is an indispensable component for scaled-up BESs. In this study, a PMS, including a power supply, power branch converter, signal transmission box, waterproof connector, and process monitoring/control system was developed for regulating and monitoring each individual electrode in the BES (Fig. 3). Each EM was first connected to a monitoring unit that contained a load (i.e., resistor, R < 50 mΩ) and Ag/AgCl reference electrode (saturated KCl, +197 mV vs standard hydrogen electrode). By measuring the voltage across the resistor, we could calculate the current that was generated by each individual cell. Moreover, the reference electrode allowed us to monitor the potential of each electrode. To address the demand for controlling multiple EMs, we also used a hub to connect the different monitoring units to the power supply unit. The power supply unit was used here to create potential differences between the anodes and cathodes of each individual cell to facilitate the growth of the electrode-active biofilm and encourage the flow of electrons.

### 2.4 EM assembly

**Fig. 4:**
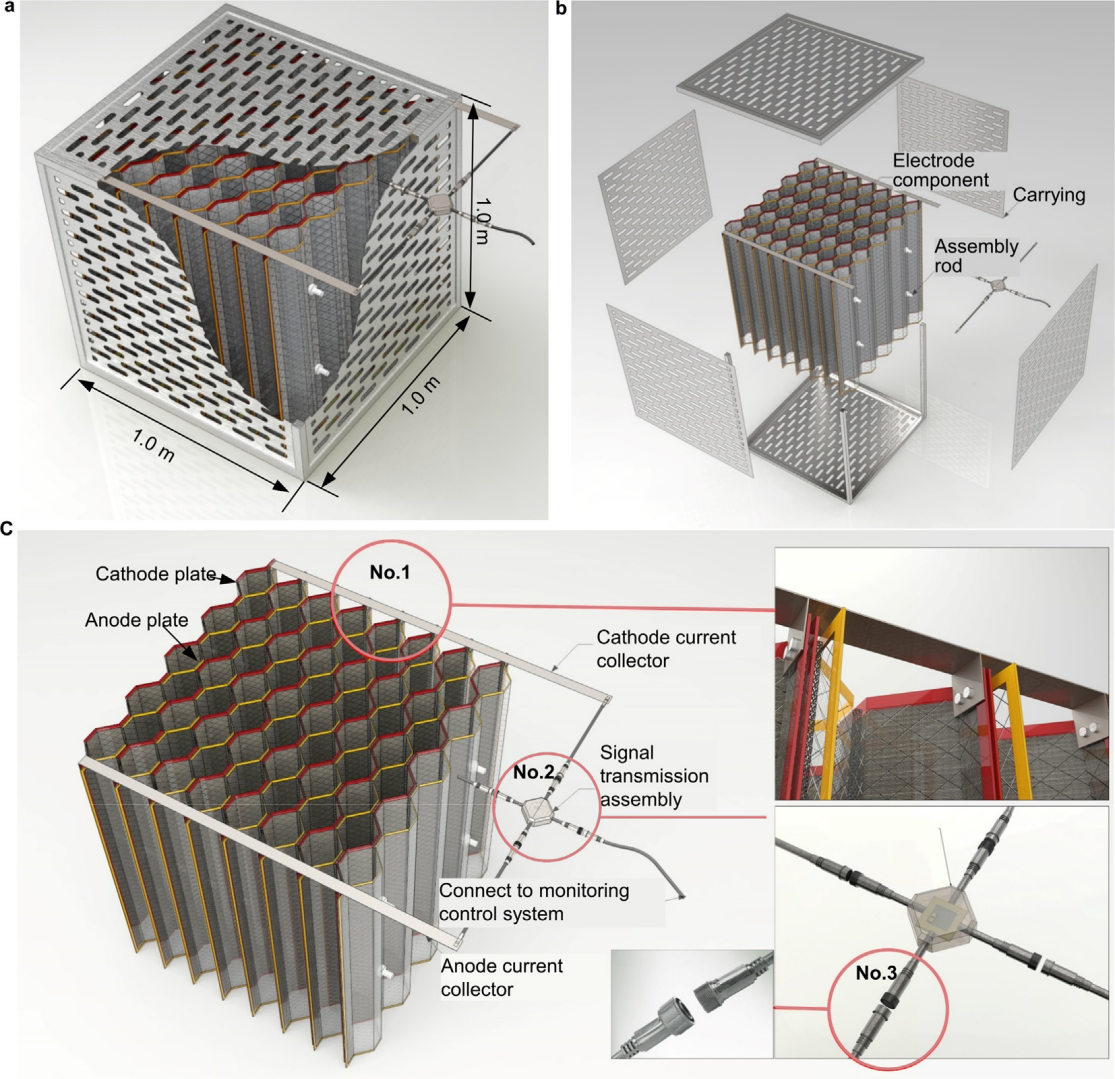
(a) conceptual global diagram of standard applicable electrode module (volume of 1 m^3^ per unit); (b) disassembly diagram of applicable electrode module; (c) partial enlargement diagram, No.1 red circle: connection method of electrode and current collector, No.2 red circle: signal transmission assembly, No.3 red circle: waterproof connector.

The EM can operate as a moveable and extendable environmental bioremediation facility. It consisted primarily of three parts (electrode component, PMS, and carrying component) with a total volume of 1 m^3^ (L × W × H: 1.0 m × 1.0 m × 1.0 m) (Fig. 4). The EM consisted mainly of eight pairs of sandwich-corrugated electrodes, a current collector, PMS, and unplasticized polyvinyl chloride (UPVC) cage.

### 2.5 Economic analysis

As a novel and promising stimulated environmental bioremediation technology, all the improvements in the electrode module manufacture or reactor configuration thus far discussed must be compatible with the target of low manufacturing costs if we wish to make this technology economically feasible [17]. Numerous studies regarding the techno-economic analysis of electrodes or BESs for practical application were conducted to certify the economic feasibility [41,42]. Most importantly, the capital costs of electrochemistry-stimulated technology must be acceptable to target the field of large-scale wastewater treatment [43].

**Fig. 5:**
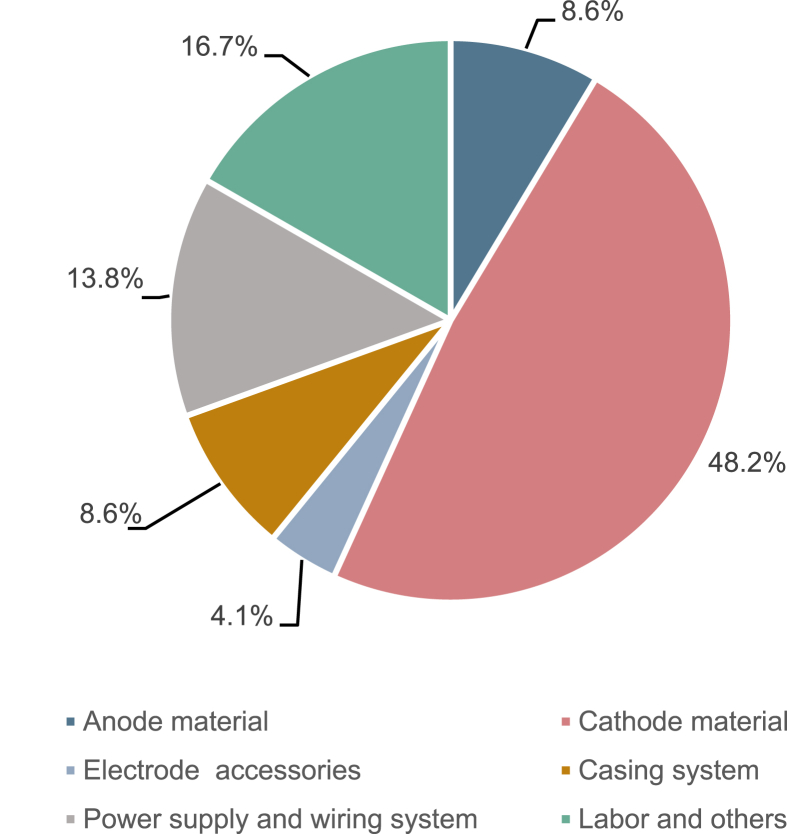
Decomposition of capital costs of proposed electrode module.

Fig. 5 provides an overview of the estimated capital costs of an applicable EM based on the material and manufacturing cost. The unit cost of this standard applicable EM equipment was estimated to be 595.7 $/m^3^ (Table S1). The raw material cost of the anode and cathode accounted for the main parts (56.8%), including the carbon felts ($235.7) and corrugated SS mesh electrode plate ($102.6). Other accessories for the anode and cathode, such as current collector, isolation pads, protection parts, and screws used for the EM cost 4.1% of the total cost. The casing system containing the assembly rod and PVC casing shell accounted for 8.6% of the total cost ($51.20). The power supply and wiring system accounted for 13.8% of the total cost, including the signal transmission assembly, waterproof connector and wire, DC power supply, and voltage branch divider. However, this part was not considered in the previous study. Table 1 compares the anticipated costs of the applicable EM with the capital costs of the previously reported BES modules. This comparison indicates that based on the liter-scale of the system or electrode, the capital costs of a full-scale BES were estimated to be in the large range 735–36000 $/m^3^, based on the different configuration and design. The low cost of BESs or EMs was mainly attributed to the separator membrane; the BESs with a capital cost less than 2000 $/m^3^ did not include a membrane or used an inexpensive substitution membrane. The high costs of those systems with a cost greater than 10000 USD·m^-3^ were attributed the high cost of the membrane. The EM in this study was superior in terms of low operation cost and easy maintenance. Moreover, in the previous study, the capital costs of BES built-in EM was compared with other wastewater treatment technologies (such as up-flow anaerobic sludge bed (UASB), anaerobic sequencing batch reactor (ASBR), anaerobic fluidized-bed reactor (AFB) and up-flow constructed wetland (UFCW)) which achieve the same effect to removal the azo dye. The results (Table S2) demonstrated that BES built-in EM shows comparable or even lower capital cost of other wastewater treatment technologies.

**Table 1:** Comparison of capital costs among the scaling-up microbial electrochemical electrode.

System description	Scale	Substrate	Electrode materials	Capital costs	Membrane	Reference
Electrode modules	1 m^3^	Wastewater	Stainless steel/carbon felt	735$/m^3^	None	This work
Microbial electrochemical system	1.5 m^3^	municipal wastewater	graphite fiber brushes	936$/m^3^	Dynamic membrane	[15]
Single chamber microbial fuel cells	0.002 m^3^	domestic wastewater	Cathode: stainless steel mesh Anode: Hard felt	$1543/m^3^	None	[75]
Microbial fuel cells	22.5 m^3^	municipal wastewater	Anode: carbon cloth Cathode: carbon cloth	2133$/m^3^	PEM	[76]
Microbial fuel cells	22.5 m^3^	municipal wastewater	Anode: carbon cloth Cathode: Pt-based carbon cloth	2225$/m^3^	PEM	[76]
Microbial electrolysis cell	0.175 m^3^	domestic wastewater	stainless steel mesh	$9622/m^3^	None	[77]
Tubular microbial fuel cells	0.2m^3^	municipal wastewater	Anode: carbon brush; cathode: carbon cloth	11126$/m^3^	IEM	[14]
Cubic-type microbial fuel cells	1m^3^	municipal wastewater	granular activated carbon	36000$/m^3^	CEM	[78]

## 3 Performance of integrated process for anaerobic wastewater treatment

### 3.1 Integration strategies

The integration of BES and conventional anaerobic treatment processes was proved to be a promising strategy for enhancing pollutant degradation during wastewater treatment. Arguably, the manner in which the BESs are combined with the conventional anaerobic treatment processes is the single most important issue, determining the overall performance of the combined system. To determine the effect of the relative positions, we conducted a series of studies to investigate the relative volumetric proportion and acclimatization strategies of the EM with respect to pollutant-removal performance.

In terms of the relative position of the BESs within the anaerobic process, our previous study suggested that placing the BES in the liquid phase of an anaerobic reactor is more beneficial to the removal of pollutants, compared to placing the BES in the sludge phase [44]. This can be attributed to the following. Firstly, the amount of pollutants can be degraded in the anaerobic sludge phase preventing the possible biotoxicity inhibition of the pollutants on the electrode microbe. Secondly, computational fluid dynamics (CFD) simulation has demonstrated that electrodes installed in the liquid phase can concentrate on the middle part of the up-flow region and somewhat ignore the cross-sectional area, which can provide a 2–3 times greater water flow velocity in the electrode zone, thus improving the mass transport to the electrode surface and potential pollutant removal in the EMs [45]. In terms of the relative volumetric proportions, our study demonstrated that the optimal volumetric proportion of EMs and sludge should be less than one, i.e., the total volume occupied by the EMs should be less than the sludge volume to achieve superior pollutant degradation performance [46]. In terms of acclimatizing the electrode active biofilm, our previous studies have indicated that the acclimation of nonspecific functional electrodes is critical for the integrated system to allow treatment of complicated electron-acceptor coexisting wastewaters [[47], [48], [49]]. Online and offline acclimation were the two primary methods to startup the EMs in an integrated system. Adjusting the external applied voltage and inoculating electrochemically active bacteria were the most common used acclimation methods for the integrated system startup. Furthermore, the “polarity inversion protocol” was proposed for rapid acclimation of the nonspecific functional electrodes. This protocol proposes establishing a bioanode first based on the diverse reductases and electron transfer-related proteins of the anode-respiring bacteria in a reduced time (approximately 12 days), and then switching the bioanode to the cathode directly to accelerate refractory contaminant (such as azo dye and nitroaromatic) reduction [50].

### 3.2 Performance of full-scale case study

**Fig. 6:**
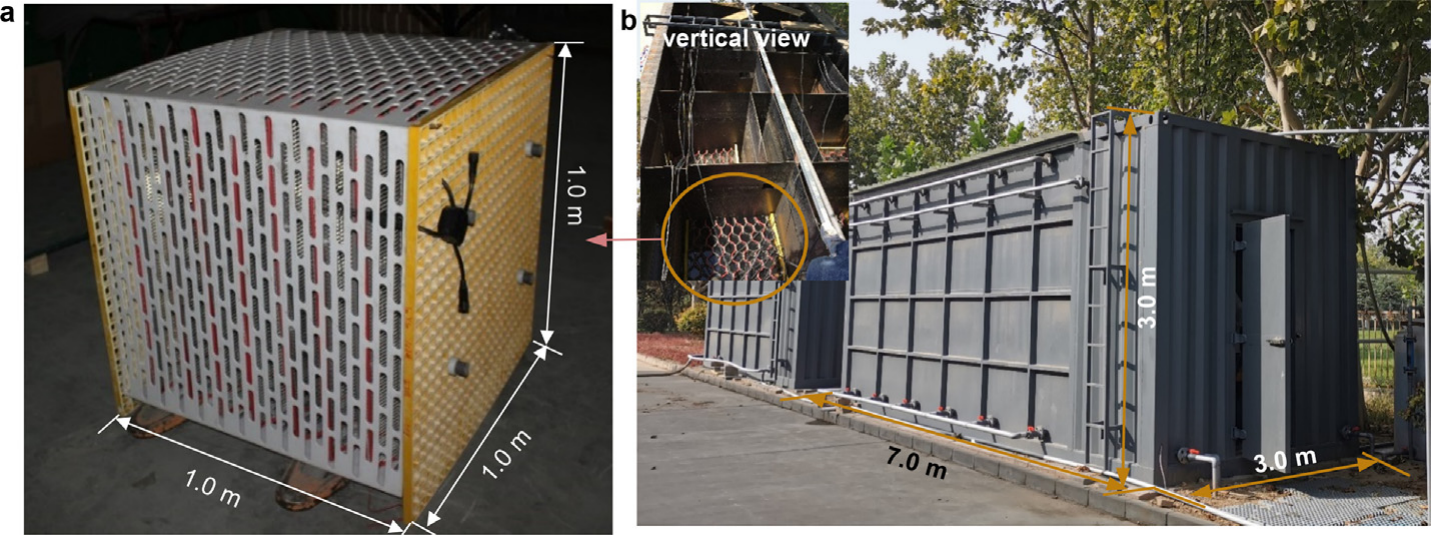
(a) photograph of sandwich-corrugated electrode module; (b) photograph of pilot-scale reactors with sandwich-corrugated electrode module.

The promising results from both the liter-scale and scaling-up tests encouraged us to further test the proposed corrugated-sandwich EMs in larger BESs, with the goal of better evaluating and optimizing the design of the proposed EMs. Hence, we constructed a hybrid treatment process with 14 cubic-meter-scale corrugated-sandwich EMs integrated into a traditional anaerobic treatment process, to accelerate the treatment of effluents from a pharmaceutical industrial park (Fig. 6). The full-scale hybrid anaerobic reactor design was based on an anaerobic baffle reactor (ABR, length 7 m × width 3 m × height 3 m) into six tanks with the total working volume of 45 m^3^. The hybrid anaerobic reactors contained 14 standard applicable EMs (1 m × 1 m × 1 m), each tank with two standard EMs arranged up and down. The feed was pharmaceutical wastewater pretreated in a primary sedimentation tank (COD: 156–363 mg/L, BOD_5_: 52–108 mg/L, ammonia: 21.8–49.9 mg/L, TN: 28.1–60.7 mg/L, SS: 182–381 mg/L, color: 512–2048 times). The inoculation sludge was from the wastewater treatment plant and the MLVSS of the hybrid-anaerobic reactor was maintained at approximately 5000 mg/L. The HRT of the system was 20 h and it was operated with an influent temperature of 25–35 °C. During the start-up period, the external voltage of was on-line regulated by the PMS from 0.3 to 0.7 V which controlled the anode potential was lower than 200 mV. Current output was observed in all EMs in 2 days and finally got to the plateau after going through a rising duration. The maximal current density attained was 1.25 ± 0.11 A/m^2^ at a voltage of 0.7 V. The current generation errors between the 14 EMs were within 18.7% which demonstrated that these EMs with a good stability even in the full-scale application. Moreover, *Geobacter* and *Shewanella* species were observed in the anode biofilms of EMs demonstrated that the EMs facilitated the microbial growth. During about six months’ operation, the COD and color removal efficiency of the EM based BESs were observed as 35.2 ± 12.8% and 67.2 ± 21.4%, respectively, which enhanced 16.7 ± 13.2% and 59.4 ± 27.9% compared with the original treatment process (without BESs).

## 4 Future perspectives of electrode module application

**Fig. 7:**
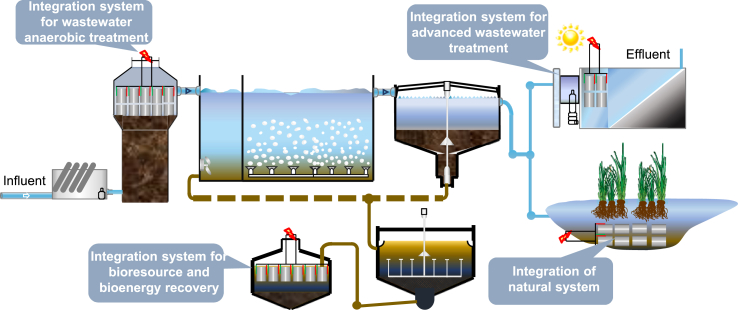
Potential scenarios wherein sandwich-corrugated EMs could be used to incorporate BESs with existing wastewater treatment technologies.

BES as an innovative option for simultaneously environmental bioremediation and resource/energy recovery as the increasing environmental concerns and resource/energy crisis. In this study, we demonstrated the feasibility of constructing scaled-up BESs using the proposed newly developed sandwich-corrugated EMs. Although our demonstration studies were conducted in stand-alone BESs, we believe that the proposed sandwich-corrugated EM could also allow us to incorporate BESs with different existing mainstream technologies for improved pollution control and bioresource recovery (Fig. 7). Besides aforementioned considerations for the standard EM construction, other aspects such as the surface modification of electrode materials, multi-module application mode, maintenance and recyclability need to be considered while engineering electrodes for different BES application scenarios.

### 4.1 Integration system for anaerobic wastewater treatment

It is not uncommon that wastewater treatment plants are tasked with the demands of treating high-strength wastewater or recalcitrant pollutant removal, especially for industrial wastewaters. In such situations, the incorporation of BESs could significantly enhance the treatment capability of the traditional anaerobic wastewater treatment processes. As we have demonstrated in lab-scale studies, BESs could be placed at the liquid phase of an upflow anaerobic sludge reactor. In terms of reactor configuration, EMs are typically placed entirely or partially in the liquid phase of the anaerobic treatment reactor for enhancing and polishing the stream from the anaerobic sludge zone, as the bioelectrochemicals are more suitable for treating medium to low-strength wastewaters with a highly biodegradable composition [51,52], whereas the anaerobic sludge has a competitive advantage in addressing high-strength wastewaters. The particulates and complex substrates could then be hydrolyzed and fermented at the bottom sludge phase into easily biodegradable substrates (such as volatile fatty acids, glucose) [44,45]. Subsequently, these substrates could be degraded by electrode-active bacteria associated with the BES in the upper part of the reactor [53]. More importantly, the incorporated BES could also remove certain recalcitrant pollutants through an electrochemical reduction process, which could allow the treatment facilities to meet the more stringent discharge standards.

### 4.2 Integration system for bioresource and bioenergy recovery

The proposed EMs could also be utilized to treat high-strength waste streams for bioenergy/bioresource recovery. In terms of bioenergy recovery, the proposed modules could allow the construction of a built-in microbial electrolysis cell within traditional anaerobic digesters for CH_4_/H_2_ recovery [38,[54], [55], [56]]. Because the extent of the enhanced bioenergy recovery through BES is largely dependent on the current density of the installed BES, it is therefore of significant importance to have EMs that are in favor of electron transfer, from both between the bacteria and electrodes, and among the microorganisms within the electrochemical-active biofilm [57]. Hence, we believe that the proposed newly developed EMs do possess the properties to enhance the current density of installed BESs for enhanced bioenergy recovery.

We should mention here that in addition to CH_4_ and H_2_ recovery [58,59], the proposed EM could also allow us to recover valuable N and P from high-strength wastes. For example, struvite (MgNH_4_PO_4_·6H_2_O) precipitation is an appealing approach for phosphate, ammonia, and magnesium recovery from wastewater simultaneously [60,61]. By deploying a BES into a struvite-precipitation reactor, the struvite precipitation efficiency could be improved near the cathode owing to higher pH values [62]. For the nitrogen recovery, the module-based BES could allow a high concentration of ammonia to be efficiently recovered by coupling the BES with a recovery unit (e.g., NH_3_ stripping and membrane-based absorption). A single chamber sleeve-type reactor with applicable electrode module could facilitate the ammonia recovery of large-scale facilities. In electrode modules, catholyte can transfer from one cathode bucket to the next in series for concentrating ammonia nitrogen. A higher recovery efficiency of ammonia nitrogen could be realized by the combination of an EM and cathode bucket. Moreover, “bioelectrochemical ammoniation” (BEA) [63] showed that electroactive bacteria convert nitrate and nitrite directly to ammonium via dissimilatory nitrate reduction to ammonium (DNRA), and ammonium was accumulated as a viable nitrogen source for recovery. So the use of module-based BES in nutrient recovery has double benefits of both nitrate conversion and ammonium recovery.

### 4.3 Integration system for wastewater advanced treatment

The EM could also be integrated with advanced wastewater treatment technologies (such as membrane filtration and photocatalysis technologies) to enhance the removal of recalcitrant substances. A photo-bioelectrochemical system (PBES) coupled with an EM with a traditional photocatalysis system represents a promising system for the enhanced removal of pollutants while simultaneously converting organic wastes and solar energy into electricity [64]. In fact, a PBES with a biocathode and photoanode has been successfully integrated to remove refractory organics and nitrate; the biocathode serves as an electron donor and provides a habitat for microbial growth in the system [65,66]. Further studies are required to investigate the application of EMs in PBESs, such as the preparation of photoanodes and biocathodes, arrangement of the EMs, matching of the light source system (intensity, distance, method of irradiation), function of the biocathode in the aspect of simultaneous carbon removal, nitrification, and denitrification.

A bioelectrochemical-membrane integrated system (BES-MF) is another efficient and energy-saving technology for advanced wastewater treatment [67,68]. The proposed EM could provide a low cost, applicable, and high-operational sustainability solution for the construction of a BES-MF. The membrane technology provides sufficient biofilm development on the cathode, enables a high utilization of the oxygen as an electron acceptor at the cathode, and ensures acceptable effluent quality [67,69]. Moreover, the MFC offers the promise of partially offsetting the energy consumption in the membrane bioreactor (MBR) by generating electricity, and thus enables a more sustainable wastewater treatment. For the wide application of the applicable electrode modules in a BES-MF for advanced wastewater treatment, further studies must be explored, such as the operation modes of the electrode and membrane, optimization of the electricity-generation capacity, electrode materials, and biofilm-driven catalysis mechanisms.

### 4.4 Integration system for natural systems

A constructed wetland (CW) is a well-recognized environmentally benign process for low-strength wastewater treatment. A recent study demonstrated that a CW can be integrated with BESs (BES-CW) for synergistical pollutant removal, bioelectricity production, reduction of greenhouse gases, and removal of heavy metals [70]. The feasibility of the integrated system (electrode module and constructed wetland) is based on the oxidation-reduction potential gradient in the vertical of the CW (especially the vertical subsurface type). The installed electrode module in the CW could provide sufficient electron acceptors/donors, and thus obtain a high removal efficiency of pollutants (i.e., organic, nitrogen, and sulfur), even at a low C/N ratio, which has the net effect of simultaneously mitigating greenhouse gas (N_2_O and CH_4_) emission [71]. The implementation of EMs to the CW could also enhance the biodegradation of emerging organic containments (EOCs, i.e., p-chloronitrobenzene, antibiotics) to alleviate the inhibition of organic and nitrogen removal, even under reduced temperatures. Furthermore, although the level of current derived from electrode module in the BES-CW is substantially less than the commercial application standard, it has the potential to be indirectly used as a biosensor as it can reflect the bioactivities and conditions of the microbes [72,73]. Although the electrode module has demonstrated its superior performance and benefits, for a wider application, the previously discussed research topics require further study.

Bioelectroremediation is an efficient, sustainable, and environment-friendly remediation technology for contaminated sediments [74]. The proposed EM provides a potential option to directly control the operation parameters during the in situ degradation of pollutants in sediments. Matrix-arranged engineered electrodes could facilitate the synergistic removal of conventional and characteristic contaminants from river sediments and overlying water. The overlying water area could also be combined with an EM and ecological floating island to fully exert the combined remediation function of electrochemistry, microorganisms, and plants, and finally achieve the purification of the polluted water ecological environment.

## 5 Conclusion

Developing EMs that are engineering-wise plausible and economically viable for scaled-up systems is the greatest hurdle in developing BESs that can be applied in solving real-world problems. In this study, we demonstrated that a novel design of an EM consisting of a carbon felt and SS mesh electrode, PMS, and other accessory systems could enhance the performance of different biological waste treatment technologies. With proper engineering, the EMs not only have a superior flow regime on the macrometer level, but also provide a favorable condition for electrode active bacteria colonization and electron transfer on the micrometer level. The performance of integrating a system of electrode modules and anaerobic reactors for enhancing industrial wastewater treatment from liter-scale to cubic-meter scale demonstrated promising results in terms of enhancing the performance of traditional biological wastewater treatment technologies. We believe that the proposed EM could provide a viable option to deploy scaled-up BESs in existing wastewater treatment facilities for improved pollution control. Moreover, we also envision that the proposed EM could have ramifications in other bioremediation areas by enabling researchers in other fields to integrate BES into other treatment technologies.

## Declaration of competing interest

We declare that we have no financial and personal relationships with other people or organizations that can inappropriately influence our work, there is no professional or other personal interest of any nature or kind in any product, service and/or company that could be construed as influencing the position presented in, or the review of, the manuscript entitled.
