# Supplementary material for: Electrochemistry-stimulated environmental bioremediation: Development of applicable modular electrode and system scale-up
Source: Environ Sci Ecotechnol. 2020 Jun 26;3:100050. doi: 10.1016/j.ese.2020.100050 (PMC9488061; doi:10.1016/j.ese.2020.100050)
Supplement: Multimedia component 1 [file mmc1.docx]

**Supplementary material**

**Electrochemistry-Stimulated Environmental Bioremediation: Development of Applicable Modular Electrode and System Scale-up**

Ai-Jie Wang ^a,b,*^, Hong-Cheng Wang^a^, Hao-Yi Cheng^a^, Bin Liang^b^, Wen-Zong Liu^b^,

Jing-Long Han ^a^, Bo Zhang^b^, Shu-Sen Wang ^b^

*a. School of Civil & Environmental Engineering, Harbin Institute of Technology (Shenzhen), Shenzhen 518055, PR China*

*b. Key Laboratory of Environmental Biotechnology, Research Center for Eco-Environmental Sciences, Chinese Academy of Sciences, Beijing 100085, P. R. China.*

*Corresponding author: waj0578 @hit.edu.cn (Ai-Jie Wang)

**Table S1 The material and mass distribution of a unit electrode module**

|  | Component | Material | Used amount/m^3^-EM | Unit price/$ | Total cost/$ |
| --- | --- | --- | --- | --- | --- |
| electrode | Anode | Graphite coating stainless-steel | 16.38 kg | 3.13 | 51.3 |
|  | Cathode plate | Graphite coating stainless-steel | 16.38 kg | 3.13 | 51.3 |
|  |  | Carbon felt | 9.6m^2^ | 28.4 | 235.7 |
|  | Current collector | Titanium | 0.05 kg | 29.4 | 1.5 |
|  | Isolation pads | rubber | 10.80 kg | 0.57 | 6.2 |
|  | Others (protection parts, screws) | Plastics/ rubber | 7.87 kg | 2.12 | 16.7 |
| Casing system | Assembly rod | PVC | 3.65 kg | 2.87 | 10.5 |
|  | Casing | PVC | 9.55 kg | 4.26 | 40.7 |
| wiring system | Signal transmission assembly | PVC/rubber | 1 unit | 14.2 | 14.2 |
|  | Waterproof connector and wire | PVC/rubber | 1 unit | 21.3 | 21.3 |
|  | DC power | plastics, cooper and iron | 1 unit | 32.6 | 32.6 |
|  | voltage branch | PVC/rubber, Titanium | 1 unit | 14.2 | 14.2 |
| Labor and others | - | - | - | 99.5 | 99.5 |

**Table S2 Comparison of electrode module based BES and conventional anaerobic technologies when serving for azo dye removal**

| Target removal efficiency (%) | conventional anaerobic technologies | | | | | electrode module based BES |
| --- | --- | --- | --- | --- | --- | --- |
|  | anaerobic  process | HRT/h | Co-substrate | Capital cost  /€·kgAO7·d^-1^ | References | Capital cost  /€·kgAO7·d^-1^ |
| 80 | UASB | 24 | sucrose,0.588 g.L^-1^ | ***708.3*** | [1] | ***656.9*** |
| 81 | ASBR | 24 | sucrose | ***1049.4*** | [2] | ***700.7*** |
| 90 | AFB | 24 | - | ***1.89*×10^5^** | [3] | ***1426.6*** |
| 92 | UASB | 24 | acetate | ***1523.3*** | [4] | ***1709.9*** |
| 94 | UFCW | 72 | C_6_H_5_COONa &CH_3_COONa, 0.41g.L^-1^ | ***2712.8*** | [5] | ***2038.9*** |
| 98 | UASB | 79.2 | glucose,2g.L^-1^ | ***3148.1*** | [6] | ***2653.5*** |
| 98 | UFCW | 72 | C_6_H_5_COONa &CH_3_COONa, 0.41g.L^-1^ | ***5204.2*** | [7] | ***2653.5*** |

1. Ong, S.-A., et al., *Decolorization of azo dye (Orange II) in a sequential UASB–SBR system.* Separation and Purification Technology, 2005. **42**(3): p. 297-302.

2. Ong, S.-A., et al., *Decolorization of Orange II using an anaerobic sequencing batch reactor with and without co-substrates.* Journal of Environmental Sciences, 2012. **24**(2): p. 291-296.

3. Seshadri, S., P.L. Bishop, and A.M. Agha, *Anaerobic or aerobic treatment of selected azo dyes in wastewater.* Waste Management, 1994. **14**(2): p. 127-137.

4. Bras, R., et al., *Monoazo and diazo dye decolourisation studies in a methanogenic UASB reactor.* J Biotechnol, 2005. **115**(1): p. 57-66.

5. Ong, S.A., et al., *Treatment of azo dye Acid Orange 7 containing wastewater using up-flow constructed wetland with and without supplementary aeration.* Bioresour Technol, 2010. **101**(23): p. 9049-57.

6. Mendez-Paz, D., F. Omil, and J.M. Lema, *Anaerobic treatment of azo dye Acid Orange 7 under fed-batch and continuous conditions.* Water Res, 2005. **39**(5): p. 771-8.

7. Ong, S.A., et al., *Simultaneous removal of color, organic compounds and nutrients in azo dye-containing wastewater using up-flow constructed wetland.* J Hazard Mater, 2009. **165**(1-3): p. 696-703.
